# Simultaneous Exposure of Cultured Human Lymphoblastic Cells to Simulated Microgravity and Radiation Increases Chromosome Aberrations

**DOI:** 10.3390/life10090187

**Published:** 2020-09-10

**Authors:** Sakuya Yamanouchi, Jordan Rhone, Jian-Hua Mao, Keigi Fujiwara, Premkumar B. Saganti, Akihisa Takahashi, Megumi Hada

**Affiliations:** 1 Gunma University Heavy Ion Medical Center, Maebashi, Gunma 371-8511, Japan; 3ra9di8o.y@gmail.com; 2 Radiation Institute for Science & Engineering, Prairie View A&M University, Prairie View, TX 77446, USA; jrrhone@pvamu.edu (J.R.); pbsaganti@pvamu.edu (P.B.S.); 3 Biological Systems & Engineering Division, Lawrence Berkeley Laboratory, Berkeley, CA 94720, USA; jhmao@lbl.gov; 4 Department of Cardiology, University of Texas MD Anderson Cancer Center, Houston, TX 77030, USA; KFujiwara1@mdanderson.org

**Keywords:** lymphoblast, microgravity, space radiation, chromosome aberration

## Abstract

During space travel, humans are continuously exposed to two major environmental stresses, microgravity (μ*G*) and space radiation. One of the fundamental questions is whether the two stressors are interactive. For over half a century, many studies were carried out in space, as well as using devices that simulated μ*G* on the ground to investigate gravity effects on cells and organisms, and we have gained insights into how living organisms respond to μ*G*. However, our knowledge on how to assess and manage human health risks in long-term mission to the Moon or Mars is drastically limited. For example, little information is available on how cells respond to simultaneous exposure to space radiation and μ*G*. In this study, we analyzed the frequencies of chromosome aberrations (CA) in cultured human lymphoblastic TK6 cells exposed to X-ray or carbon ion under the simulated μ*G* conditions. A higher frequency of both simple and complex types of CA were observed in cells exposed to radiation and μ*G* simultaneously compared to CA frequency in cells exposed to radiation only. Our study shows that the dose response data on space radiation obtained at the 1*G* condition could lead to the underestimation of astronauts’ potential risk for health deterioration, including cancer. This study also emphasizes the importance of obtaining data on the molecular and cellular responses to irradiation under μ*G* conditions.

## 1. Introduction

Astronauts on deep-space missions will experience continual microgravity (μ*G*) and uninterrupted doses of ionizing radiation higher than any exposure experienced in the history of human spaceflight. It is predicted that, during the 3-year human mission to Mars, astronauts will be simultaneously exposed to altered gravity and chronic radiation, with a cumulative effective radiation dose of about 1 sievert (Sv) [1]. The types of radiation were found in the space environment, such as galactic cosmic rays (GCR) and solar particle events (SPE). The space radiation is more damaging to cells [2]. The biological impact of simulated space radiation on mammalian cells and rodents has been extensively investigated in ground-based experiments. Such studies have shown that space radiation not only has higher cancer risk [3,4], but also negatively affects the function of the cardiovascular [5,6], central nervous [7,8] and immune [9,10,11] systems. The effects of μ*G* on mammalian cells have been studied extensively on the International Space Station (ISS), or by using clinostats for ground-based experiments. It is well known that μ*G* affects various cellular functions such as altered cell adherence, protein expression, gene expression, morphology, increased apoptosis and decreased proliferation rates [12,13,14,15].

In space biology studies, there is a basic question; what is the effect of exposing cells simultaneously to μ*G* and space radiation? The answer to this question has significant implications on the astronauts’ health risk assessment and management during long-term space missions. There are many investigations about the combined effects of space radiation and μ*G*, carried out both in space and on the ground (see the review by Moreno-Villanueva et al.) [16]. Unfortunately, there is no clear consensus. Some reported synergy (i.e., increased detrimental effects), but some did not. Performing experiments in space is challenging since there is a limited number of opportunities (if any) to repeat the same experiment under the same condition. The sequential treatments, such as cells being irradiated and then exposed to simulated μ*G*, have been used in some ground-based experiments. In these experiments, cells are not exposed to radiation and μ*G* at the same time. There are only a few cases in which cells were irradiated while being under the simulated μ*G* condition. However, for the experiments of this type, it was often necessary to stop clinostat rotation in order to irradiate cells. Recently, we have developed a ground-based system for irradiating cells while they are under simulated μ*G* using a three-dimensional (3D) clinostat [17,18] as a new system for ground-based space environment studies. This system allows the irradiating of cells without stopping the rotation of the clinostat, hence it is truly simultaneous exposure. As the clinostat rotates, the platform on which cell culture chambers are mounted becomes perpendicular to the direction of the irradiation beam every minute. Cells are irradiated for 0.2 s with either X-ray or carbon ion (C-ion) beam. For X-ray irradiation, a high-speed shutter located in front of the beam port opens for 0.2 s. For C-ion bean irradiation, a respiratory gating system for heavy ion treatment is used.

Using these experimental set-ups, we have earlier studied the combined effects of radiation and μ*G* on human fibroblasts, which are adherent cells. We found that the expression of cell cycle-suppressing genes decreased, and that of cell cycle-promoting genes increased after C-ion irradiation under μ*G* compared with irradiation [19]. The cell cycle may pass through the checkpoints with DNA damage due to the combined effects of C-ion and μ*G*, suggesting that increased genomic instability might occur in space [19]. Indeed, we also found an increased frequency of chromosome aberrations (CA) in cells exposed to both radiation and μ*G*, compared to those treated by radiation only [20].

In this work, we studied the combined effects of simulated μ*G* and irradiation on human lymphoblastic TK6 cells, which were originally isolated from spontaneous immortalized lymphoblastic WIL2 cells [21]. TK6 cells are relatively stable and express the wild-type tumor suppressor *p53* gene [22]. Unlike fibroblasts that are adherent cells, TK6 cells grow in suspension. TK6 cells are commonly used in radiation studies to investigate genomic instability [23,24,25] and known to be highly radiosensitive due to efficient radiation-induced apoptosis [26]. The endpoints measured in this study were cell survival and CA.

## 2. Materials and Methods

### 2.1. Cell Culture

Cells were obtained from ATCC (Manassas, VA, USA). Since the experiment was performed outside the CO_2_ incubator, cells were cultured in CO_2_-independent medium (COI) (Thermo Fischer Scientific, Waltham, MA, USA) containing 10% (*ν*/*ν*) fetal bovine serum (MP Biomedicals, Santa Ana, CA, USA), 200 mM L-glutamine (Thermo Fischer Scientific) and 1% (*ν*/*ν*) penicillin–streptomycin mixed solution (Nacalai Tesque, Kyoto, Japan), and kept at 37 °C. We also used RPMI medium (Thermo Fischer Scientific) containing 10% (*ν*/*ν*) fetal bovine serum (MP Biomedicals), 1% (*ν*/*ν*) penicillin–streptomycin mixed solution (Nacalai Tesque) and 10 mM-HEPES (Dojindo Molecular Technologies, Inc., Kumamoto, Japan) at 37 °C in a CO_2_ incubator for checking cellar growth. We used disposable, sealed irradiation cell culture chambers (DCC) (Chiyoda Co., Kanagawa, Japan) [27,28]. Exponentially growing cells were seeded into DCC.

### 2.2. Simultaneous Exposure of Cells to Radiation and Simulated μG

A system for subjecting cultured cells simultaneously to radiation and simulated-μ*G* (SSS) using a 3D clinostat (PMS-CST I, Advanced Engineering Services Co. Ltd. (AES), Ibaraki, Japan) has been previously described [20]. Cells were irradiated under the simulated μ*G* condition without stopping the clinostat motion. We used an X-ray generator (200 kV, 14.6 mA, aluminum filter (0.3 mm thick), MultiRad225: Faxitron Bioptics, LLC, Tucson, AZ, USA), and a high-speed shutter (Accelerator Engineering Co., Chiba, Japan) placed in front of the X-ray irradiation port for synchronized X-ray irradiation. For synchronized C-ion irradiation, a synchrotron (Gunma University Heavy Ion Medical Center, Gunma, Japan) and respiratory gating systems were used. The dose-averaged linear energy transfer is 50 keV/μm at the center of the 6 cm spread-out Bragg peak of the beam, with an energy of 290 MeV/n [29]. Cells were irradiated by a 0.2 s pulse of beam when the cell chamber on the clinostat stage becomes perpendicular to the direction of irradiation beam, which took place once every minute. The X:Y ratios of clinorotation were set at 11:13 rpm and = 66°/s:78°/s, to accurately synchronize irradiation when the samples were in a horizontal position. For the control, cells in the chamber were mounted on a stationary clinostat to achieve static condition (1*G*), with the same pulse irradiation [17,18,29,30]. The dose rate was approximately 0.03 Gy/min for both X-ray and C-ion irradiation under the simulated μ*G* or 1*G* conditions. Peltier modules were loaded on the 3D clinostat and the static stage, to maintain constant temperature at 37 °C [17].

**Figure 1:**
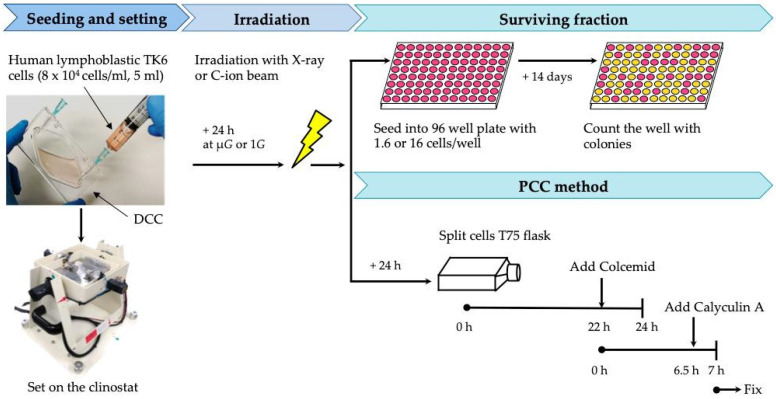
Scheme of experimental flow for cell survival and chromosome aberrations (CA) studies. Cells grown in disposable, sealed irradiation cell culture chambers (DCC) cassettes were mounted on the clinostat and exposed to X-ray or C-ion, with or without rotating clinostat.

Figure 1 shows the details of experimental flow. Frozen TK6 cells were revived and cultured for 4 days prior to seeding into DCCs at the concentration of 8 × 10^4^ cells/mL. The chamber was set on the 3D clinostat. Cells were on the clinostat (or the stationary clinostat) for 24 h prior to irradiation. The cells were then irradiated with X-ray or C-ion under μ*G* condition without stopping clinostat rotation. For survival fraction analysis, cells were harvested immediately after irradiation, in order to observe the effect of radiation in the cells’ exposure under μ*G* or 1*G* conditions. Regarding CA, cells were kept under μ*G* or 1*G* conditions for 24 h after irradiation, to observe the unrepaired DNA damages.

### 2.3. Cell Survival Fraction Analysis

After irradiation, TK6 cells were collected from DCCs and seeded into 96-well plates at various densities depending on the dose and type of radiation, which affected cell survival differently. For X-ray irradiation cells, seeding density was 1.6 cells/mL. For C-ion irradiation, cells were seeded with 1.6 cells/mL for 0.25 Gy, 16 cells/mL for 1.0 Gy, and densities were variable for 0.5 Gy and 0.75 Gy. The plates were incubated for 10–14 days at 37 °C. The wells with the culture medium color change from red to yellow were defined as having colonies, while those with no-color change were defined as no colony formation. We calculated plating efficiency (PE) and survival fraction using the following formula.

PE = (−ln (EW/TW))/N
(1)

where EW is the number of wells with colonies, TW is total number of wells, and N is the average number of cells initially plated per well.

Survival fraction (%) = (PE of treatment group)/(PE of control group) × 100
(2)

where the treatment group is exposed to radiation and μ*G* simultaneously, the control group is exposed to radiation only.

Three 96 well-plates were made for each irradiation scheme (X-ray stationary, X-ray clinostat, C-ion stationary, C-ion clinostat), 288 well for each scheme were analyzed.

### 2.4. Premature Chromosome Condensation (PCC)

We used the PCC technique to collect G2/M-phase chromosomes as previously described [31,32,33]. After irradiation, TK6 cells were kept for 24 h under static or μ*G* conditions, to allow them to repair damaged DNA. Then, the cells were split into T75 flask at low density and subcultured at 37 °C. After incubation for 22 h, we added KaryoMAX^®^ Colcemid^®^ solution (Thermo Fisher Scientific) to the final concentration of 90 ng/mL in the culture medium, to arrest the cells in mitosis, and incubated them for an additional 7 h before harvesting. Approximately 30 min before harvesting, 50 nM of Calyculin A (FUJIFILM Wako Pure Chemical Co., Osaka, Japan) was added to the culture, so that chromosomes in the G2 phase in the cell cycle would be condensed.

### 2.5. Fluorescence In Situ Hybridization (FISH)

CA was analyzed as previously described [20,34]. Chromosomes were spread on slides and hybridized in situ with a combination of three fluorescence whole-chromosome human DNA probes for chromosomes 1 (red), 2 (green) and 4 (yellow) (Aquarius, Cytocell, Oxford Gene Technology, Oxfordshire, UK), according to the recommended protocol by the manufacturer. Then, we used DAPI (4,6-diamidino-2-phenylindole) to stain all chromosomes. The complex exchange was defined as two or more chromosomes containing three or more breaks [32]. The simple exchange was defined as an exchange of arms between two chromosomes, resulting in dicentrics and translocations. Incomplete translocations and incomplete dicentrics were classified as simple exchanges, assuming that, in most cases, the reciprocal fragments are below the level of detection. Each type of exchange (dicentrics, apparently simple reciprocal exchanges, incompletes, or complex) was counted as one exchange, and values for total exchanges were derived by adding the yields. When two or more painted chromosomes were damaged, each was scored separately. At least 1000 cells were scored or headed up to 50 aberrations for each data point in the sample of each irradiation dose.

### 2.6. Statistical Analysis

We calculated the ratio between aberrations scored and total cells analyzed for the frequencies of CA as reported previously [20]. Several studies have indicated that the distribution of radiation damages among chromosomes is random, and the yield of exchanges measured within the first division after exposure is proportional to the DNA content of the chromosome analyzed, with some fluctuation of data [35]. We extrapolated the frequencies of exchanges in individual chromosomes to whole-genome equivalents using the following formula reported by Lucas et al. [36].

Fp = 2.05[fp(1 − fp) + fp1fp2 + fp1fp3 + fp2fp3]FG
(3)

where Fp is the combined frequency of exchanges in all painted chromosomes; fp is the fraction of the whole genome comprised of the painted chromosomes; fp1, fp2 and fp3 are the fractions of the genome for chromosomes 1, 2 and 4, respectively; and FG is the whole-genome aberration frequency. From this formula, the genomic frequency for TK6 cells was calculated to be 2.48 times that detected on chromosomes 1, 2 and 4.

Standard errors for aberration frequencies were calculated assuming Poisson statistics. Error bars in the figure represent the standard error of the mean values. The Fisher’s exact test was used to assess whether the difference in frequency of chromosome exchanges is significant between different treatment groups. Logistic regression analysis was performed to examine the effect of radiation dose and gravity on total exchanges, using total exchange as dependent variable and radiation dose and gravity as independent variables.

## 3. Results

### 3.1. Cell Survival

**Figure 2:**
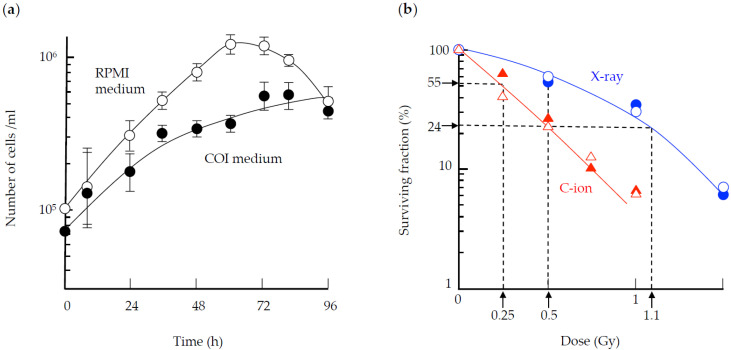
Cell growth and survival. (**a**) The growth of TK6 cells cultured in RPMI medium (white) or in CO_2_-independent medium (COI) (black) is shown. The error bar indicated the standard error of the mean. n = 3 for each time point. (**b**) Survival fraction of TK6 cells irradiated by 0.2 s pulses of X-ray (blue circle) or 290 MeV/n C-ion (red triangle) under the μ*G* (unfilled) or 1*G* (filled) condition. Experimental data represent the mean of two to three plates for each dose experiment.

Figure 2a shows the growth of TK6 cells cultured in regular RPMI medium in a CO_2_ incubator and COI medium. Compared to the RPMI medium, cell growth was slower in the COI medium. Under the culturing condition using the COI medium, the doubling time was 24 h. Figure 2b shows the survival fraction irradiated by X-ray and 290 MeV/n C-ion under the static condition. Since cells were irradiated by 0.2-s pulses under simulated μ*G* and at 1*G* static condition, the survival study was conducted using the 0.2-s pulse exposure, as well as continuous exposure. After maintaining μ*G* or 1*G* condition for 24 h and irradiation, the unirradiated cell numbers of 1*G* condition were 1.28 × 10^5^ ± 0.222 cells/mL, while the unirradiated cell numbers under μ*G* condition were 1.17 × 10^5^ ± 0.354 cells/mL. For each dose level, the cumulative dose of pulse irradiation was the same as that of continuous exposure. There was no significant difference in the survival fraction between μ*G* and 1*G*. Compared to the survival curve for cells irradiated by X-ray which showed a shoulder, the exponential survival curve for C-ion had no such shoulder. C-ion was more lethal than X-ray per unit dose.

Under the pulse irradiation mode, a total dose of 0.5 Gy C-ion beam exposure and 1.1 Gy X-ray exposure resulted in similar cell survival. We also found that 0.25 Gy C-ion beam exposure and 0.5 Gy X-ray exposure had similar cell survival fraction. Based on these results, we selected the cumulative doses of 0.5 Gy, 1.1 Gy and 1.5 Gy for X-ray, 0.25 Gy and 0.5 Gy for C-ion, to assess the combined effect of simulated μ*G* and radiation on CA in TK6 cells.

### 3.2. CA

**Figure 3:**
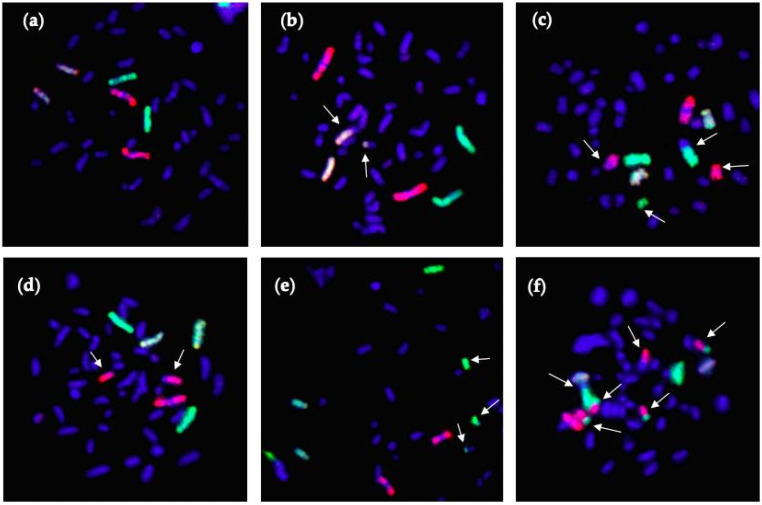
Examples of 3-color whole-chromosome FISH staining images of TK6 cells: chromosome 1 (red), chromosome 2 (green) and chromosome 4 (yellow). CA are identified by arrows as simple (reciprocal exchanges between two chromosomes) or complex-type exchanges (three or more breaks in two or more chromosomes). (**a**): normal chromosome spread; (**b**): simple type of exchange between chromosome 4 and another chromosome (dicentrics); (**c**): simple type of exchange between chromosome 1 and another chromosome (incomplete exchanges) and chromosome 2 and another chromosome; (**d**): fragmentation of chromosome 1; (**e**): complex type of exchange between chromosome 2 and other chromosomes; (**f**): complex type of exchange involving chromosomes 1, 2 and another chromosome.

Figure 3 shows 3-color chromosome FISH images, in which chromosome 1 (red), chromosome 2 (green) and chromosome 4 (yellow) are stained. All chromosomes were stained by DAPI (blue). Figure 3a shows normal chromosome with no damages. Other panels show chromosomes that are damaged in various ways (see figure legend). All types of detectable aberrations in chromosomes 1, 2 and 4 were scored, and the whole-genome equivalent frequencies of aberrations were calculated.

Table 1 shows the total exchanges analyzed in this study. Chromosomes 1, 2 and 4 consist of roughly 40% of the nuclear DNA. The frequencies of CA obtained from chromosomes 1, 2 and 4 were converted to the whole nuclear DNA equivalent.

**Figure 4:**
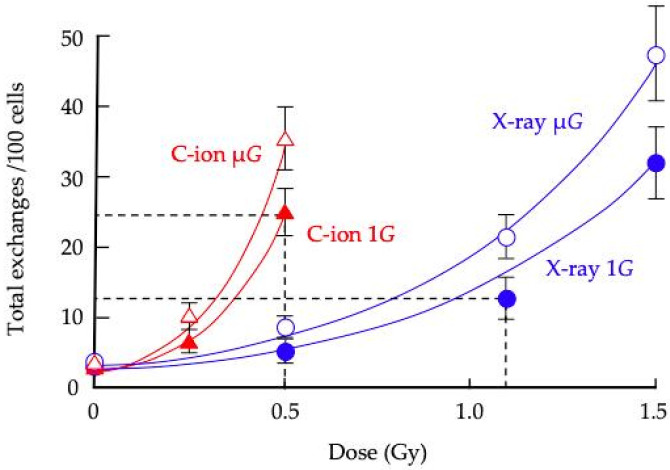
Frequencies of total chromosome exchanges induced by X-ray (blue circle) or C-ion beam (red triangle), while cells were under either 1*G* (filled) or simulated μ*G* conditions (unfilled). Error bars indicated the standard error of the mean. “n” for each data point is shown in Table 1.

Figure 4 shows the relationship between irradiation doses and total exchanges. For each source of radiation, the frequency of CA increased with dose. The frequency of CA increased in cells exposed under the μ*G* condition compared to those exposed under the static condition. At the same irradiation dose, CA was more frequently observed in C-ion irradiated cells than in X-ray irradiated counter parts. Furthermore, at the iso-survival fraction dose, C-ion irradiation produced more CA than X-ray irradiation.

Table 2 shows the logistic regression analysis of the effect of radiation dose and gravity on total exchanges. The results indicate that both dose and gravity significantly contribute to total exchanges.

**Figure 5:**
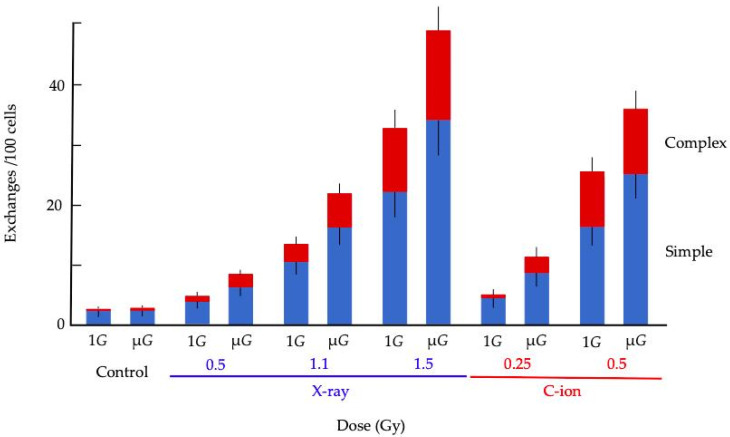
Frequencies of simple and complex types of chromosome exchange induced by X-ray or C-ion beam while cells were under either 1*G* or simulated μ*G* conditions. The blue column shows a simple type of CA and the red column shows a complex type of CA. Error bars indicated the standard error of the mean value. “n” is shown in Table 1.

Figure 5 shows the more detailed analysis for CA in TK6 cells irradiated with X-ray and C-ion beam. The blue bars indicate the simple type of CA, and the red bars represent the complex type of CA. The frequencies of CA were increased dose-dependently by both irradiation types. The proportion of complex CA was similarly increased. The rate of increase in frequency was higher for C-ion irradiation than X-ray irradiation.

## 4. Discussion

Humans will face the unique environment in space, represented by both μ*G* and the space radiation, which contains a wide variety of ion species with various ranges of energy. It is challenging to simulate these space environments in one’s laboratory. Although many studies have reported the increased sensitivity of cells to radiation and decreased levels of DNA repair under simulated μ*G*, most of these studies were performed using X-ray or γ-ray [16]. In the future Mars missions, GCR exposure, made up of high-energy and charged nuclei, will be of more serious concern than low linear energy transfer (LET) exposure. For this reason, it is important to investigate the effects of both low- and high-LET exposure under the μ*G*. In our previous studies, we have shown changes in cell cycle-related genes expression [19] and increased frequency of CA [20] in human fibroblasts using our unique SSS [17,18].

CA data obtained from chromosome 1, 2 and 4 were extrapolated to the whole genome using a modified version of the equation of Lucas et al. [36]. Originally, Lucas reported the formula to calculate whole- genome exchange frequency based on low-LET radiation exposure. Although damages caused by high-LET exposure were expected to different form damages induced by low-LET exposure, we applied this modified formula in many cases of single-mono energetic ion beams in various LET, as well as mixed beam [33,37,38], and the results were in good agreement with the CA frequencies simulated by Monte Carlo modeling both single beam and mixed beams [39,40,41]. This formula is applicable to high-LET exposure in the range of doses we used in this study.

CA has been shown to increase in the lymphocytes of astronauts after long missions lasting for several months in space [42,43,44]. CA is positively correlated with increased incidents of cancer [43,44,45]. Therefore, CA is a gold standard in the biodosimetry for radiation, and has been used for the risk assessment of space radiation. To follow up our previous study with adherent 1BR-hTERT cells [20], we used non-adherent TK6 cells to investigate whether the similar combined effect of radiation and μ*G* on CA frequency is observed. Compared to the cells exposed to radiation only, higher frequencies of CA were found in TK6 cells under the condition of simultaneous exposure to radiation and μ*G* (Figure 4). Our study indicates that this combined effect is independent of radiation types, as both X-ray and C-ion beams were more detrimental under μ*G* than 1*G*. Thus, our study suggests that the effects of ionizing radiation and μ*G* are more than additive and that astronauts’ risk of CA increases during long space flights. We observed higher frequencies of CA in TK6 cells with the same dose of irradiation. Under the same survival fraction dose, we detected increased incidence of the complex type of CA in TK6 cells, although the frequency of total CA was similar between TK6 cells and 1BR-hTERT cells. It is not clear if this difference is due to higher radio sensitivity of TK6 cells than that of 1BR-hTERT or due to the way these cells are cultured: adherent and floating. It is reported that there was wide variation in chromatid break frequencies, but it was only slightly higher than that reported for primary human cells in TK6. The dicentric frequency was more than 10-fold higher for TK6 cells than that reported for normal primary human cells. TK6 cells also show much higher levels of non-reciprocal chromosome translocations than are usually observed in primary human cells [46]. The higher frequency of the complexed exchanges in TK6 compare to fibroblasts in our study supports a previous report. The extent of the combined effect might depend on the cell types. Further investigation is needed sing different cell types.

Manti et al. reported no effects of simulated μ*G* on CA in human lymphocytes irradiated with X-ray or proton beam [47], whereas Mosesso et al. showed increased CA in human lymphocytes irradiated by 1.5 Gy of X-ray [48]. Our results on both adherent [20] and floating (current study) cells support the results of Mosesso et al. [48]. More importantly, we have observed these effects, not only with X-ray exposure, but also with relatively low dose of C-ion exposure as well, which is more relevant for the condition in the ISS [49,50,51,52]. These results support the reports of increased genomic damages in human lymphoblasts exposed to ionizing radiation under simulated μ*G* conditions compared to 1*G* [53,54].

Based on the cell survival analysis, we irradiated cells with the doses that induce the same survival fraction. Compared to the cell irradiated with X-ray, higher frequencies of CA were observed with C-ion irradiation. It is well known that CA induction by heavy ions indicates a higher frequency of complex exchanged compare to low-LET radiation, and the observed rearrangements are of greater complexity [55]. The complex type of CA observed in cells irradiated with C-ion are higher compare to irradiated to X-ray both under μ*G* and 1*G* in this study. Monte Carlo simulations as well as various other experiments indicate that complex DNA lesions with multiple damage sites, including double strand breaks, single strand breaks, and other damages within one or two helical turns referred to as clustered DNA damage are caused by heavy ion irradiation [56,57]. DNA breaks by X-ray irradiation are much less clustered, and it is likely to be repaired properly. Since clustered DNA breaks are difficult to correctly repair, breaks made by heavy ion irradiation are more likely to cause CAs.

In this study, no significant difference was observed in cell survival between μ*G* and 1*G*, suggesting that the extent of all cause cell death including apoptosis is the same. Since the frequency of CAs was greatly increased by simultaneous exposure to simulated μ*G* and radiation compared to radiation alone, it is possible that cells were proliferating with unrepaired CA. For the survival fraction analysis in this study, cells were first acclimatized to the simulated μ*G* or 1*G* condition, then irradiated under the respective gravitational condition, and seeded for culturing at 1*G* immediately after irradiation (Figure 1).

The exact reasons for increased CA under simulated μ*G* have not been fully elucidated to date. Previous studies suggest that the expression of DNA repair genes was decreased in human lymphoblasts grown under simulated μ*G* [58], and that CA in human peripheral blood lymphocytes were enhanced by simulated μ*G* through inhibition of DNA repair [59]. These results appear to establish correlation between decreased expression of DNA repair genes and CA found in cells exposed to μ*G*. In our previous study, we found decreased cell cycle-suppressing gene and increased cell cycle-promoting genes on human fibroblasts grown under μ*G* and C-ion irradiation. It was suggested that cells containing DNA damages induced by the combined stimulation with μ*G* and C-ion may be allowed to rapidly pass through the cell cycle checkpoints without complete DNA repair, and that this may induce increased CA in these cells [19]. Further studies are needed to test this hypothesis.

Although comparison of simulated μ*G* and 1*G* in each dose did not have enough statistical power to show significant difference (only 3 cases sowed *p* < 0.05 in Table 1), the multivariate logistical regression analysis provided significant contributions of μ*G* in frequency of total exchanges (Table 2). Independent of radiation exposure dose, simulated μ*G* cause 1.635 and 1.487 times of total exchanges, in comparison to 1*G* under X-ray and C-ion radiation exposure, respectively (Table 2).

Taken together, our results discussed above indicate that the effects of μ*G* and radiation are more than additive. We are aware that results from in vitro studies cannot be directly applied to in vivo biological events. The ground-based simulated μ*G* does not exactly recapitulate the μ*G* in space. However, there is limited access for spaceflight experiments. It is difficult to perform a sufficient number of replicated experiments in space to establish statistical significance. Additionally, experiments on the ISS do not give a deep space radiation environment. Thus, it is important to perform ground-based experiments using various devices that simulate the simultaneous exposure of cells and animals to μ*G* and radiation. We trust that the results from such studies will be useful in assessing various risks of space travel, and eventually coming up with means of mitigation. Further investigations are in progress. Furthermore, we anticipate that our experimental results will corroborate the recent model calculations of radiation risk uncertainties [60].

## 5. Conclusions

Higher incidence of CA in cultured human lymphoblastic TK6 cells was demonstrated when the cells were exposed to radiation (X-ray and C-ion) under simulated μ*G* than when exposed to the same level of radiation only. Our findings show that the space radiation risk assessment on human health based on experiments performed at 1*G* may result in underestimating astronauts’ potential risk for cancer and other debilitating health conditions due to GCR. We found that C-ion irradiation induced a higher frequency of complex type of CA compared to X-ray irradiation at the same cell survival fraction dose. The occurrence of complex CA was higher in TK6 cells than in human fibroblasts that we used earlier in similar CA studies. Our results suggest the importance of conducting similar studies using not only different cell types and radiation doses and types, but also other end points and model systems under the combined environment of μ*G* and radiation.

## Figures and Tables

**Table 1:** Whole-genome equivalent for frequency of CA per 100 TK6 cells by X-ray and C-ion beam under 1*G* and simulated μ*G* conditions. The *p*-values of comparison of μ*G* and 1*G* in each dose were obtained by Fisher’s exact test. *p* < 0.05 is shown in bold.

Radiation	1*G* or μ*G*	Total Spreads Scored	No. of Aberrant Spreads	Simple Exchanges *	Complex Exchanges *	Total Exchanges *
Control (0 Gy)	1*G*	1049	29	2.13 ± 0.71	0.24 ± 0.24	2.36 ± 0.75
	μ*G*	1027	27	2.65 ± 0.88	0.48 ± 0.34	3.14 ± 0.87
		(*p*-value)		(0.50)	(0.62)	(0.54)
X-ray	1*G*	1034	34	4.07 ± 0.99	0.96 ± 0.48	5.03 ± 1.10
(0.5 Gy)	μ*G*	1039	39	6.44 ± 1.24	2.15 ± 0.72	8.59 ± 1.43
		(*p*-value)		(**0.022**)	(0.27)	(0.059)
X-ray	1*G*	670	50	10.36 ± 1.96	2.96 ± 1.05	13.31 ± 2.22
(1.1 Gy)	μ*G*	523	50	16.11 ± 2.76	5.69 ± 1.64	21.80 ± 3.21
		(*p*-value)		(0.70)	(0.17)	(**0.021**)
X-ray	1*G*	330	50	23.28 ± 4.18	10.51 ± 2.81	33.04 ± 4.98
(1.5 Gy)	μ*G*	253	50	34.28 ± 5.79	14.69 ± 3.79	48.97 ± 6.93
		(*p*-value)		(0.50)	(0.44)	(**0.041**)
C-ion	1*G*	1034	34	5.51 ± 1.15	1.20 ± 0.54	6.71 ± 1.27
(0.25 Gy)	μ*G*	817	49	8.49 ± 1.60	1.82 ± 0.74	10.31 ± 1.77
		(*p*-value)		(0.83)	(0.55)	(0.091)
C-ion	1*G*	435	49	16.52 ± 3.07	9.11 ± 2.28	25.63 ± 3.82
(0.5 Gy)	μ*G*	344	45	23.77 ± 4.14	10.81 ± 2.79	36.02 ± 5.09
		(*p*-value)		(0.074)	(0.71)	(0.079)

* The results are presented as mean ± S.E.M.

**Table 2:** The logistic regression analysis of the effect of radiation dose and gravity on total exchanges.

Radiation Type		Coefficient	Standard Error of Coefficient	*p*-Value	Odds Ratio	95% CI for OR *	
						Lower	Upper
X-ray	Dose	1.906	0.136	2.12 × 10^−44^	6.727	5.150	8.789
	Gravity	0.491	0.132	2.04 × 10^−4^	1.635	1.261	2.118
	Constant	−4.826	0.171	3.30 × 10^−174^			
C-ion	Dose	5.218	0.447	1.85 × 10^−31^	184.559	76.821	443.396
	Gravity	0.397	0.156	1.1 × 10^−2^	1.487	1.096	2.018
	Constant	−4.797	0.193	4.75 × 10^−136^			

* CI: confidence interval; OR: odds ratio.
